# Supplementary material for: Magnitude of venous or capillary blood-derived SARS-CoV-2-specific T cell response determines COVID-19 immunity
Source: Nat Commun. 2022 Sep 21;13:5422. doi: 10.1038/s41467-022-32985-8 (PMC9492763; doi:10.1038/s41467-022-32985-8)
Supplement: Supplementary file 1 — Supplementary Information [file 41467_2022_32985_MOESM1_ESM.pdf]

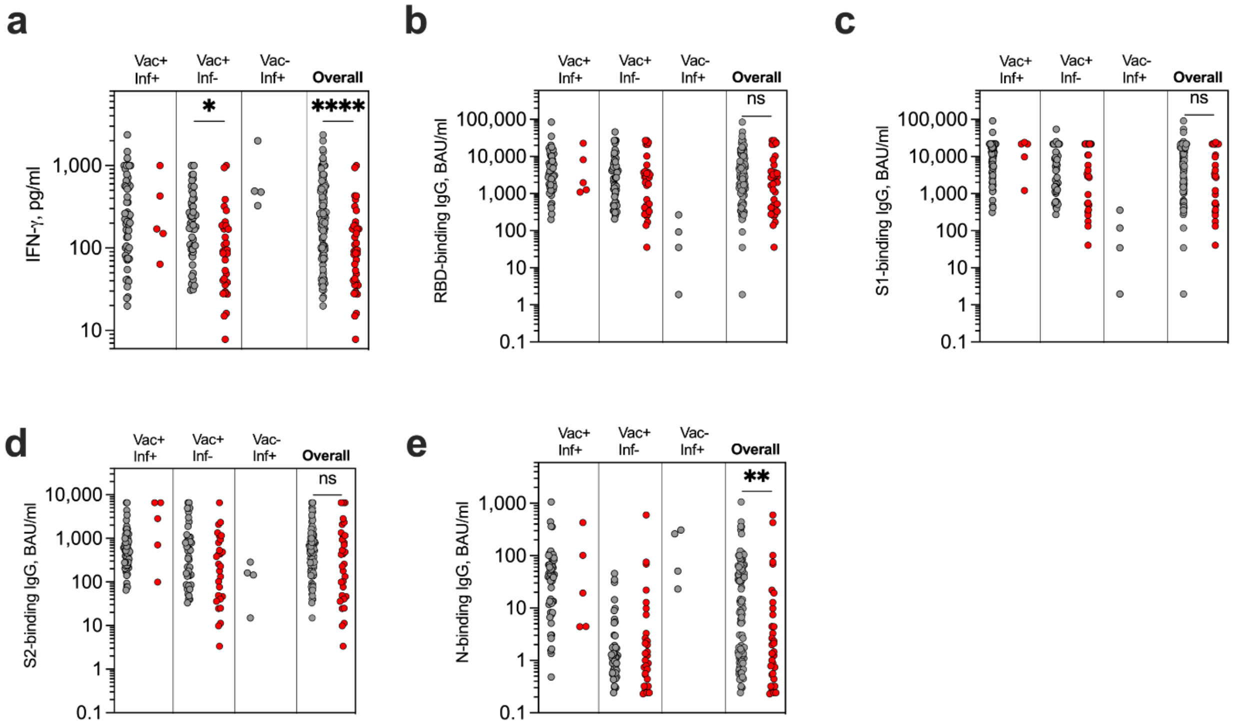

Supplementary Figure 1. Magnitude of anti-SARS-CoV-2 adaptive immune responses measured in venous blood samples up to six months preceding a positive COVID-19 test, subdivided by prior vaccination / infection status. (a) SARS-CoV-2-specific IFN-γ^{+} T cell responses were measured using the venous whole blood assay and sub-divided based on participant vaccination and prior SARS-CoV-2 (PCR and/or lateral flow test confirmed) infection status. ‘Vac+/Inf+’ n=60, ‘Vac+/Inf-’ n=82, ‘Vac-/Inf+’ n=4, ‘Vac-/Inf-’ n=1 (not plotted). **** P<0.0001, * P=0.050. SARS-CoV-2-specific IgG-binding responses targeting spike receptor binding domain (‘RBD’) (b), spike subunit 1 (‘S1’) (c), spike subunit 2 (‘S2’) (d) and nucleocapsid (‘N’) (e; ** P<0.0084) were measured using the venous whole blood assay and sub-divided based on participant vaccination and prior SARS-CoV-2 (PCR and/or lateral flow test confirmed) infection status. Participants self-reporting a COVID-19 positive test (PCR and/or lateral flow test) are highlighted (red dots); all cases of infection occurred within 6 months following blood draw.

Comparisons used Kruskal-Wallis tests with correction for multiple comparisons using Dunn's tests; comparisons of 'Overall' responses used two-sided Mann-Whitney tests. Each dot represents one donor. Source data are provided as a Source Data file.

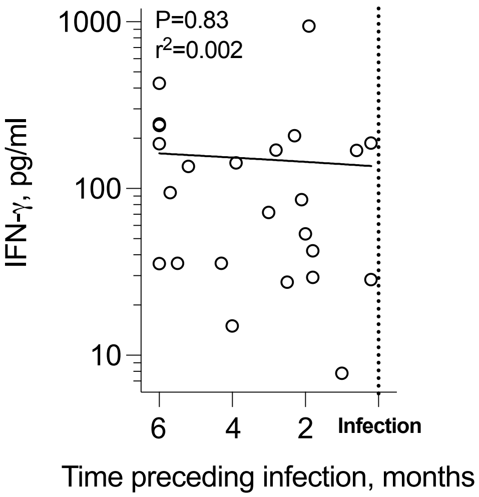

Supplementary Figure 2. Magnitude of anti-SARS-CoV-2 T cell response measured in venous blood samples prior to positive COVID-19 test. SARS-CoV-2-specific IFN-γ^+ T cell responses were measured using the venous whole blood assay. Plots indicate the magnitude of this response at the time point prior to a participant reporting a positive COVID-19 diagnostic (PCR and/or lateral flow) test. P and r^2 values indicate the results of simple linear regression with best-fit line shown. Source data are provided as a Source Data file.

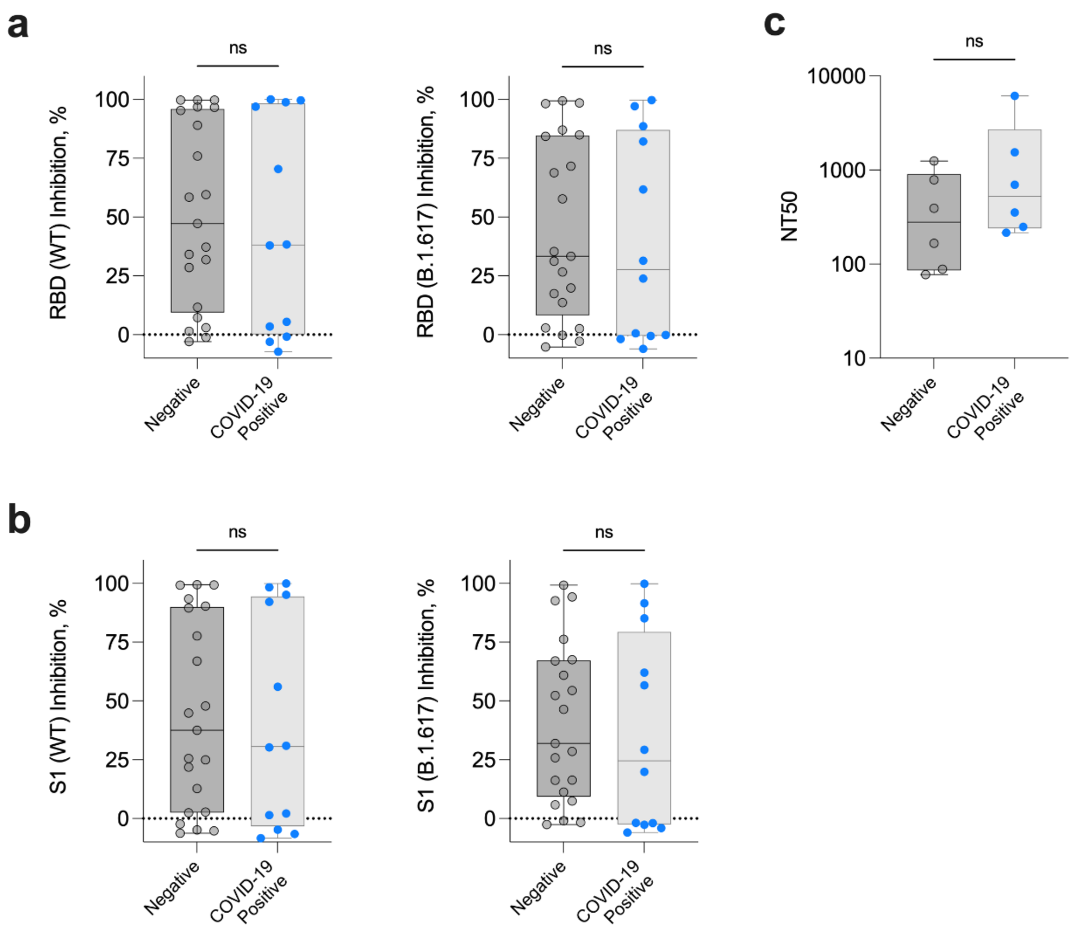

Supplementary Figure 3. SARS-CoV-2 neutralising antibody responses measured in venous blood samples up to six months preceding a positive COVID-19 test. Venous blood samples obtained from healthy donors (n=33) were assessed for neutralising antibody response specific for wild-type and SARS-CoV-2-delta (B.1.617) variant of Receptor Binding Domain (‘RBD’) (a) and Spike 1 (‘S1’) (b) subunits. Percentage inhibition data are presented as box plots (centre line at the median, upper bound at 75th percentile, lower bound at 25th percentile) with whiskers at minimum and maximum values. (c) Venous blood samples (n=12) were also assessed for neutralisation of live wild-type SARS-CoV-2 virus. Data are presented as plasma dilution required to give 50% inhibition (NT50) on box plots (centre line at the median, upper

bound at 75th percentile, lower bound at 25th percentile) with whiskers at minimum and maximum values. Each dot represents one donor. Participants self-reporting a COVID-19 positive test (PCR and/or lateral flow test) are highlighted (blue dots); all cases of infection occurred within 6 months of blood draw. Comparisons used two-sided Mann-Whitney tests. Source data are provided as a Source Data file.

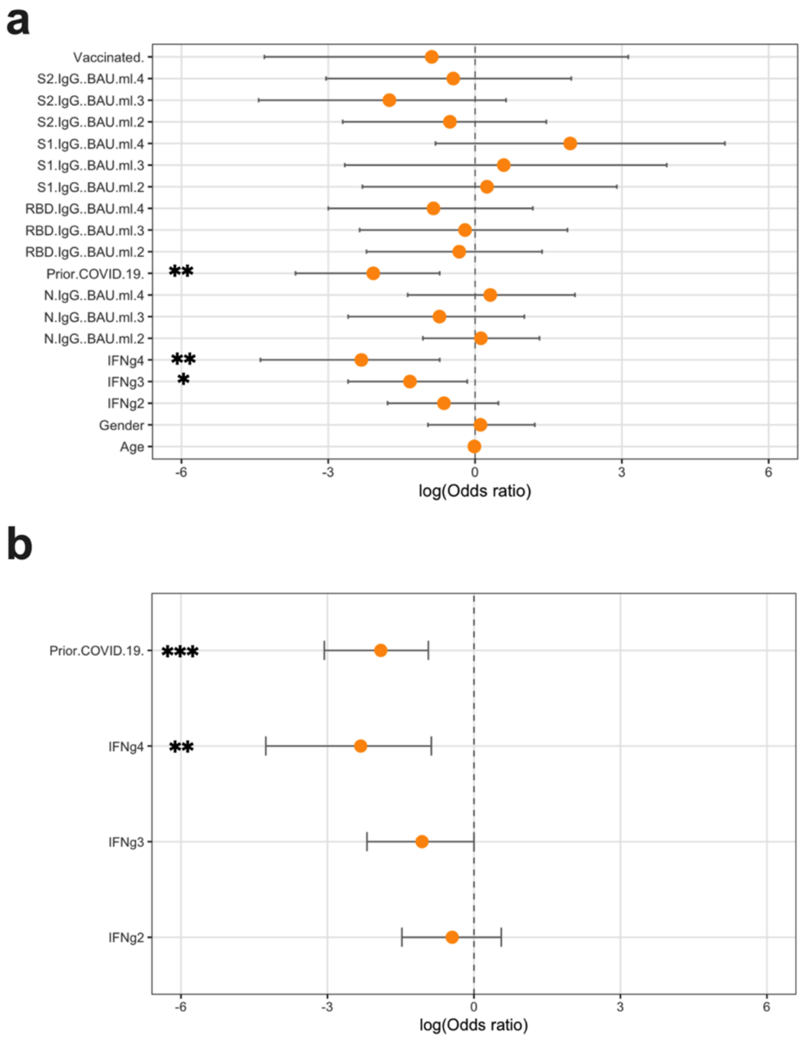

Supplementary Figure 4. Assessing parameters that influenced COVID-19 positivity. Odds ratio plot and results of binary logistic regression to model the impact of specified variables on subsequent COVID-19 positivity in the venous sampling cohort (n=148) using an initial model (a; **(Prior COVID-19) P=0.0050, **(IFNg4) P=0.0098, * P=0.031) and a cross-validated model (b; *** P=0.00035, ** P=0.0050), as detailed in the Methods. Data are shown as log(odds ratio) (orange dot) with error bars at the 95% confidence intervals.

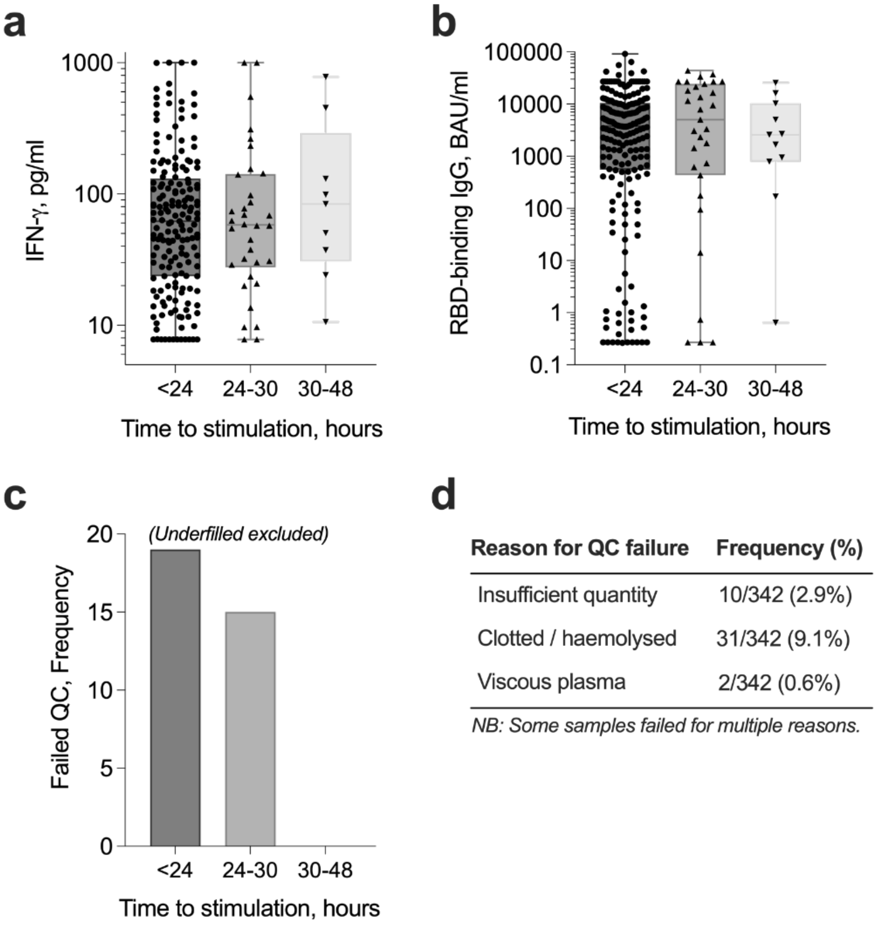

Supplementary Figure 5. Parameters affecting capillary blood measurement of SARS-CoV-2-specific adaptive immune responses. Time between capillary blood sample being obtained by the participant and stimulation with SARS-CoV-2-specific peptides, with subsequent output of SARS-CoV-2-specific IFN-γ T cell responses (a) and RBD-binding IgG responses (b), are indicated (time to stimulation <24 hours, n=177-204; 24-30 hours, n=31-34; 30-48 hours, n=9-11). Data are presented as box plots (centre line at the median, upper bound at 75th percentile, lower bound at 25th percentile) with whiskers at minimum and maximum values. Each dot represents one donor. (c) Number of samples failing quality control (QC) checks were associated with time between capillary blood sample being obtained by the participant and stimulation with

SARS-CoV-2-specific peptides. Reasons for quality control failure are indicated (d). Source data are provided as a Source Data file.

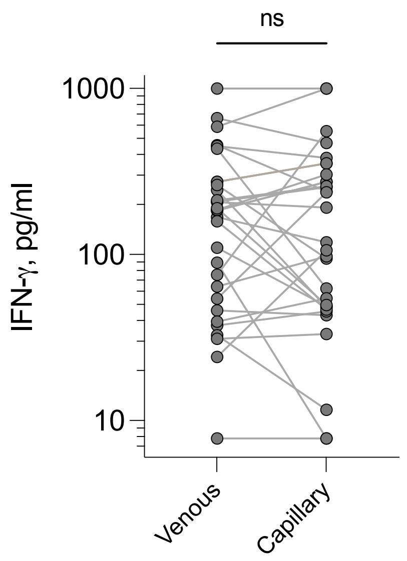

Supplementary Figure 6. Comparison of SARS-CoV-2-specific IFN-γ T cell response measurement from matched capillary and venous blood samples. The SARS-CoV-2-specific IFN-γ T cell response was measured in matched capillary and venous blood samples obtained from 31 participants. Comparison used two-sided Wilcoxon matched-pairs signed rank test (ns: not significant (P=0.8815)). Source data are provided as a Source Data file.

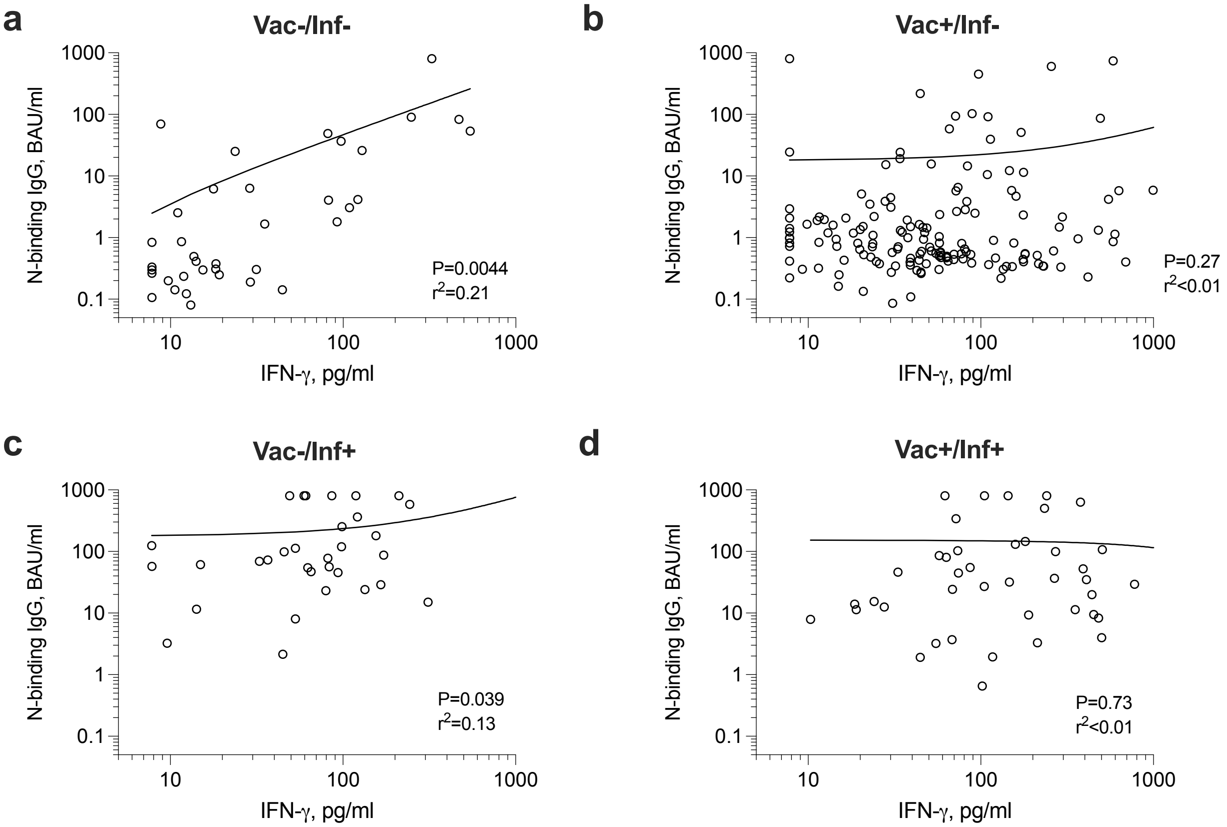

Supplementary Figure 7. Correlations between SARS-CoV-2-Nucleocapsid-binding IgG responses and SARS-CoV-2-specific IFN-γ^{+} T cell responses. Correlation between SARS-CoV-2-Nucleocapsid-binding IgG responses and SARS-CoV-2-specific IFN-γ^{+} T cell responses in capillary blood samples taken from unvaccinated participants with no prior history of infection (a), vaccinated participants with no prior history of infection (b), unvaccinated participants with confirmed prior SARS-CoV-2 infection (c) and vaccinated participants with confirmed prior SARS-CoV-2 infection (d). P and r^2 values indicate the results of simple linear regression with best-fit line shown. Each dot represents one donor. Source data are provided as a Source Data file.

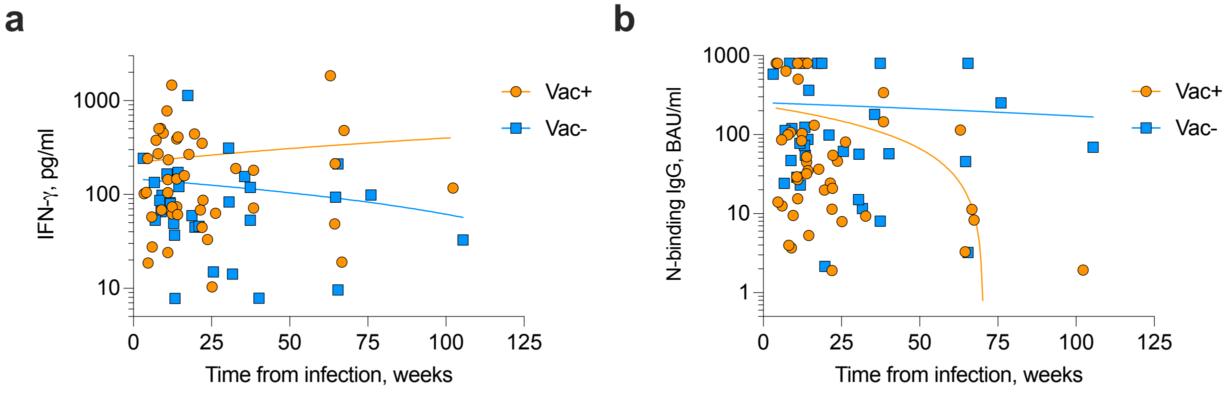

Supplementary Figure 8. Correlations between SARS-CoV-2-specific adaptive immune responses and time from infection. Participants donating a capillary blood sample and reporting prior PCR- and/or lateral flow test confirmed SARS-CoV-2 infection were sub-divided based on vaccination history (orange = vaccinated; blue = unvaccinated). Measured SARS-CoV-2-specific IFN-γ^{+} T cell responses (a) or N-binding IgG responses (b) were correlated with time from infection. Source data are provided as a Source Data file.

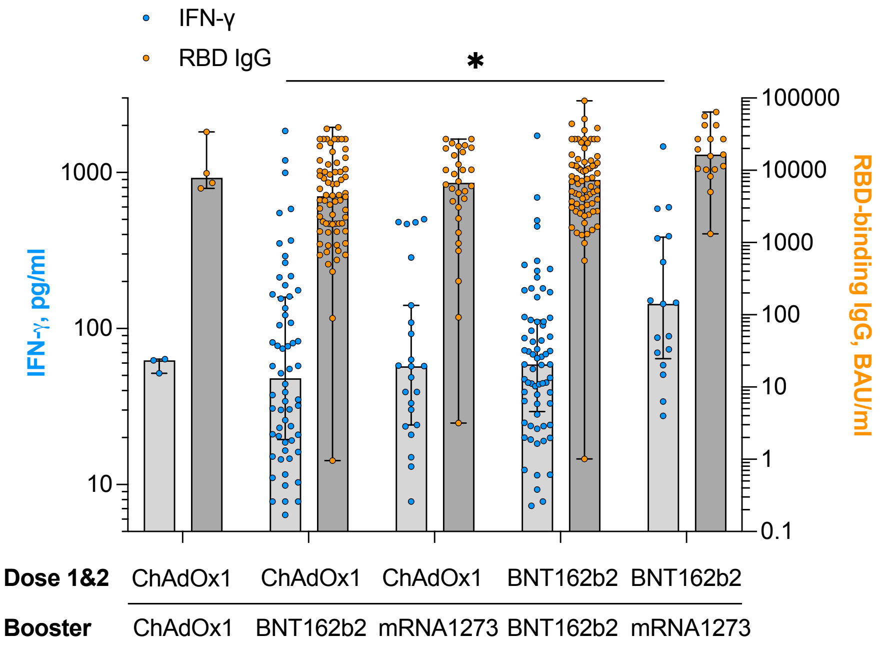

Supplementary Figure 9. Vaccination schedule and observed SARS-CoV-2-specific adaptive immune responses. Capillary blood-derived SARS-CoV-2-specific T cell (blue) and RBD-binding IgG (orange) responses were measured in vaccinated individuals, subdivided by reported vaccine type and schedule (ChAdOx1 / ChAdOx1, n=3-4; ChAdOx1 / BNT162b2, n=60-70; ChAdOx1 / mRNA1273, n=23-30; BNT162b2 / BNT162b2, n=68-74; BNT162b2 / mRNA1273, n=17). Comparisons used Kruskal-Wallis tests with correction for multiple comparisons using Dunn's tests (* P=0.033). Data are shown as median ± interquartile range. Each dot represents one donor. Source data are provided as a Source Data file.

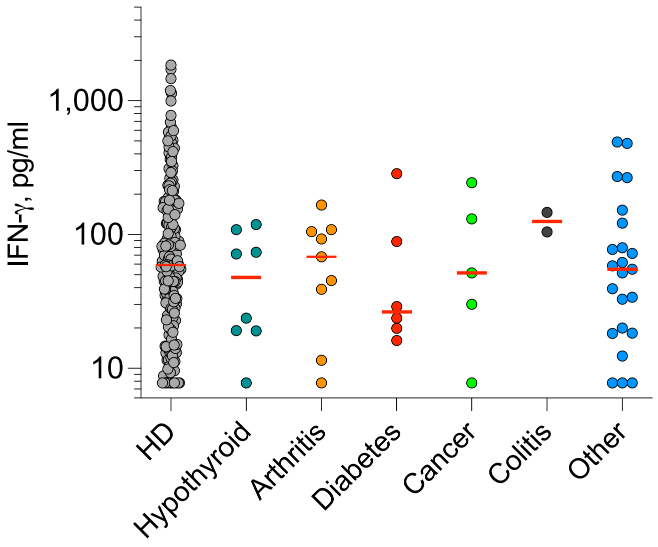

Supplementary Figure 10. SARS-CoV-2-specific IFN-γ^{+} T cell responses subdivided by reported co-morbidity. Capillary blood-derived SARS-CoV-2-specific T cell responses were measured, subdivided by reported co-morbidity (healthy donors (HD), n=218 (grey dots); hypothyroid, n=8 (teal dots); arthritis, n=9 (orange dots); diabetes, n=6 (red dots); cancer, n=5 (green dots); colitis, n=2 (black dots); other, n=23 (blue dots)). Comparisons used Kruskal-Wallis tests with correction for multiple comparisons using Dunn's tests (no significant differences observed). Red line indicates the median. Each dot represents one donor. Source data are provided as a Source Data file.

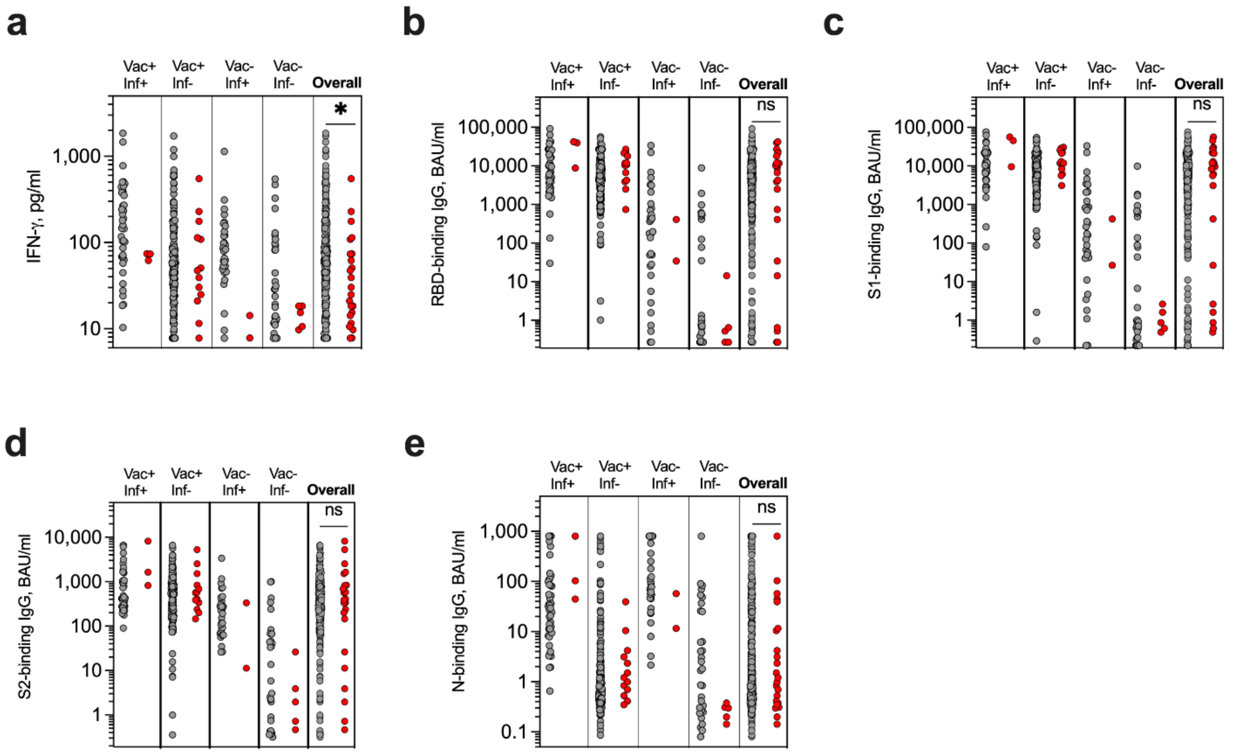

Supplementary Figure 11. Magnitude of anti-SARS-CoV-2 adaptive immune responses measured in capillary blood samples up to three months preceding a positive COVID-19 test, subdivided by prior vaccination / infection status. (a) SARS-CoV-2-specific IFN-γ^{+} T cell responses were measured using the capillary whole blood assay and sub-divided based on participant vaccination and prior SARS-CoV-2 (PCR and/or lateral flow test confirmed) infection status. 'Vac+/Inf+' n=42, 'Vac+/Inf-' n=158, 'Vac-/Inf+' n=33, 'Vac-/Inf-' n=37. * P=0.034. SARS-CoV-2-specific IgG-binding responses targeting spike receptor binding domain ('RBD') (b), spike subunit 1 ('S1') (c), spike subunit 2 ('S2') (d) and nucleocapsid ('N') (e) were measured using the capillary whole blood assay and sub-divided based on participant vaccination and prior SARS-CoV-2 (PCR and/or lateral flow test confirmed) infection status. Participants self-reporting a COVID-19 positive test (PCR and/or lateral flow test) are highlighted (red dots); all cases of infection occurred within 3 months following blood draw. Comparisons used Kruskal-Wallis tests

with correction for multiple comparisons using Dunn’s tests; comparisons of ‘Overall’ responses used two-sided Mann-Whitney tests (ns: not significant). Each dot represents one donor. Source data are provided as a Source Data file.

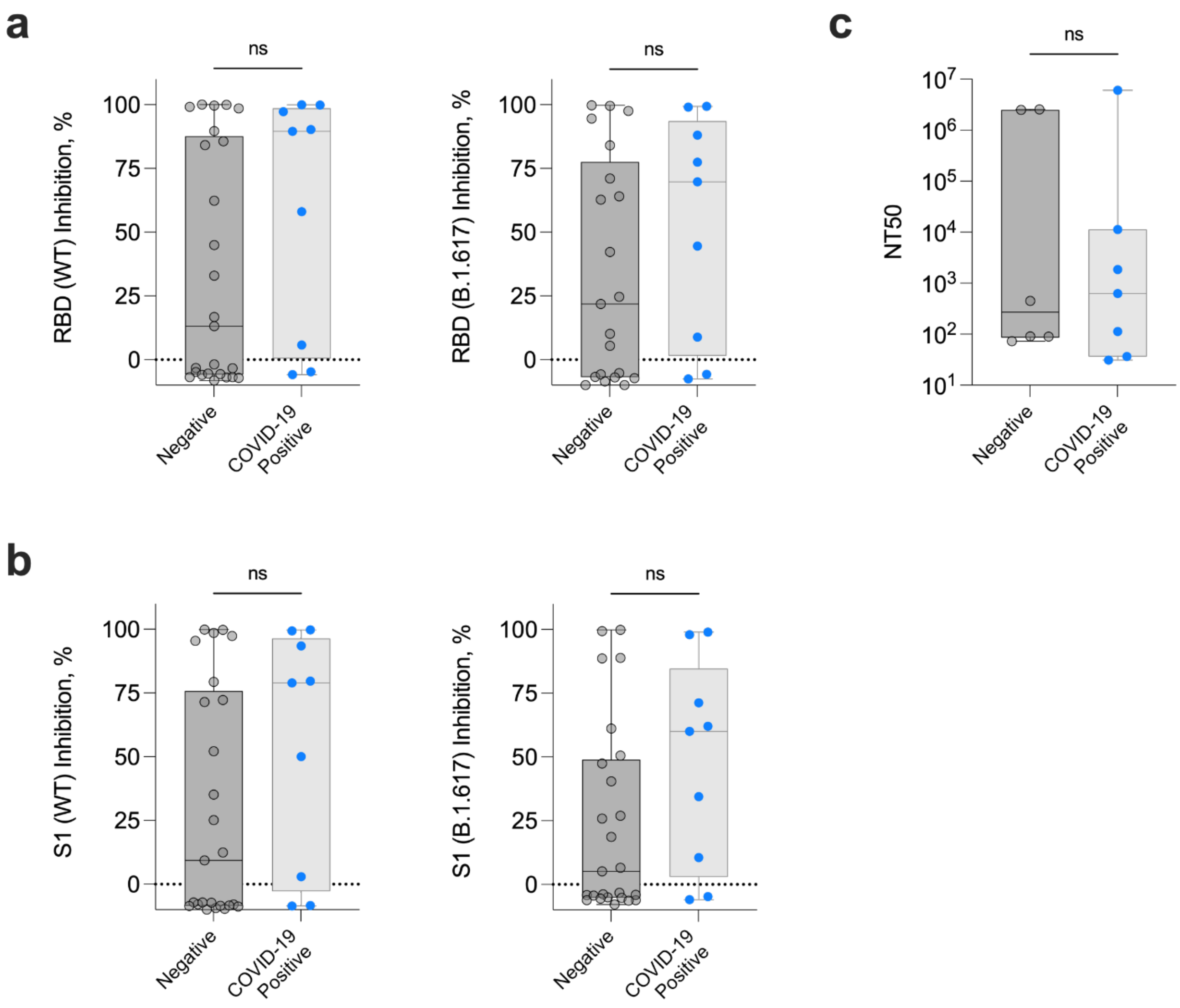

Supplementary Figure 12. SARS-CoV-2 neutralising antibody responses measured in capillary blood samples up to three months preceding COVID-19. Capillary blood samples obtained from study participants (n=34) were assessed for neutralising antibody response specific for wild-type and SARS-CoV-2-delta (B.1.617) variant of Receptor Binding Domain (‘RBD’) (a) and Spike 1 (‘S1’) (b) subunits. Percentage inhibition data are presented as box plots (centre line at the median, upper bound at 75th percentile, lower bound at 25th percentile) with whiskers at minimum and maximum values. (c) Capillary blood samples (n=13) were also

assessed for neutralisation of live wild-type SARS-CoV-2 virus. Data are presented as plasma dilution required to give 50% inhibition (NT50) on box plots (centre line at the median, upper bound at 75th percentile, lower bound at 25th percentile) with whiskers at minimum and maximum values. Each dot represents one donor. Participants self-reporting a COVID-19 positive test (PCR and/or lateral flow test) are highlighted (blue dots); all cases of infection occurred within 3 months of blood draw. Comparisons used two-sided Mann-Whitney tests. Source data are provided as a Source Data file.
